# Can AI-generated culturally contextualized listening materials enhance agentic engagement? An exploratory study with Saudi EFL students

**DOI:** 10.3389/fpsyg.2026.1861868

**Published:** 2026-07-09

**Authors:** Khalid Azzubaidi, Moteeb Alhamidani

**Affiliations:** 1 King Abdullah International Medical Research Center, Riyadh, Saudi Arabia; 2 College of Science and Health Professions, King Saud bin Abdulaziz University for Health Sciences, Riyadh, Saudi Arabia; 3 Ministry of National Guard- Health Affairs, Riyadh, Saudi Arabia

**Keywords:** agentic engagement, artificial intelligence, contextualized listening materials, cultural relevance, EFL learner

## Abstract

**Introduction:**

Previous research suggests that culturally relevant listening materials can enhance language learners' engagement. However, creating culturally contextualized listening materials requires considerable time and expertise, hindering many instructors from developing their own resources. Few studies have explored the potential of artificial intelligence (AI) in overcoming these obstacles. Addressing this gap, the present study examined the impact of AI-generated culturally contextualized listening materials on five dimensions of learner engagement among English as Foreign Language (EFL) learners in Saudi Arabia.

**Methods:**

Using a within-subjects design, 78 undergraduate students completed two AI-generated listening tasks: one relevant to Saudi culture and the other related to Western culture. Data were collected through validated post-listening questionnaires measuring behavioral, emotional, cognitive, agentic, and social engagement. Paired-samples t-tests revealed no significant differences in behavioral, social, emotional, or cognitive engagement between the two tasks.

**Results:**

However, culturally relevant materials elicited significantly higher agentic engagement (*t* = 2.70, *p* = 0.008, *d* = 0.31), suggesting that culturally contextualized materials may specifically enhance learner autonomy and proactive participation. The effect was small (*d* = 0.31) and confined to a single engagement dimension; the remaining four dimensions showed no significant differences.

**Discussion:**

The study therefore does not demonstrate a broad improvement in multidimensional engagement, and the design does not allow the agentic effect to be attributed unambiguously to cultural content as opposed to the AI-generated nature of the materials. These findings provide a preliminary indication of how AI-generated culturally situated content might inform EFL listening instruction.

## 1 Introduction

Listening comprehension is a complex cognitive process and a crucial skill in foreign language development. Research consistently demonstrates that listening is particularly challenging for English as a Foreign Language (EFL) learners to acquire, especially for learners from Middle Eastern countries such as Saudi Arabia. Saudi EFL learners often struggle with this essential skill due to multiple factors, including rapid speech rates, unfamiliar accents, and limited vocabulary knowledge. They also face other challenges due to insufficient familiarity with the content and cultural references embedded in listening materials (Al-Khresheh, 2020; Hayati, 2009). These challenges manifest in various forms of learner disengagement, including anxiety, distraction, isolation, and avoidance of listening activities, ultimately leading to poor achievement and performance (Jamal et al., 2020). The persistent difficulties Saudi learners face with listening comprehension highlight the urgent need for innovative pedagogical approaches that can enhance engagement and facilitate skill development.

Researchers in language teaching have increasingly emphasized the importance of integrating learners' cultural background knowledge into the language learning process to enhance engagement and improve learning outcomes (Al-Malki, 2018; Jamal et al., 2020; Kong et al., 2022). Cultural familiarity, which refers to learners‘ prior background knowledge of the cultural content embedded in instructional materials (Alptekin, 2006), can provide crucial contextual scaffolding, reducing cognitive load and allowing learners to focus on linguistic processing rather than struggling to understand unfamiliar cultural references. However, creating authentic, culturally relevant listening materials requires significant time and expertise in scriptwriting, audio recording, and quality assurance, which discourages many EFL instructors from developing their culturally contextualized resources.

The recent boom in artificial intelligence (AI) offers opportunities to address these issues. The application of AI in the learning environment enhances student proficiency, encourages teamwork and communication, and provides learners with more convenient access to learning resources (Qiao and Zhao, 2023; Dai and Wang, 2024). Personalized language teaching is another AI application that generates unique learning experiences tailored to individual students' needs (Nishant et al., 2020). AI-powered tools, in particular large language models (LLMs) and text-to-speech systems, have demonstrated remarkable capabilities in generating coherent, contextually appropriate content and producing speech in multiple languages (Roy and Swargiary, 2024; Woo and Choi, 2021; Yuan and Liu, 2024). Such advanced technologies would enable teachers to design culturally contextualized listening content swiftly and without advanced technical comprehension.

AI functions here as a production technology rather than a cultural agent: the cultural design of materials remains the product of human prompt engineering and review. It offers three affordances over traditional materials development: scalability, enabling rapid script generation without specialist scriptwriters (Koraishi, 2023); parametric control, allowing linguistic features to remain constant while only cultural content varies; and the capacity to embed culturally specific referential content and interactional norms when prompts are carefully designed (Koraishi, 2023; Xin et al., 2025).

Despite growing interest in AI applications for language education, research examining the impact of AI-generated culturally relevant teaching materials on learner engagement remains limited. Existing literature has devoted most of its attention to AI as a tool for feedback, assessment, or general content, and has given little consideration to culturally responsive pedagogy (Zawacki-Richter et al., 2019). Besides, a number of studies have involved engagement in language learning, but not many have considered how culturally contextualized instructional resources simultaneously affect different dimensions of engagement (behavioral, social, emotional, cognitive, and agentic).

This research study makes a significant contribution to the field. Firstly, it adds to the existing body of research on culturally responsive language teaching by emphasizing listening instruction, which has received limited research attention. Second, it provides empirical data on the use of AI-generated teaching resources for language learning, a contribution to the existing literature on AI use in EFL settings. Third, the study provides comprehensive insights into the impact of contextualized content across various dimensions of learner engagement. Lastly, the results offer practical recommendations for EFL instructors seeking scalable, efficient solutions for developing culturally relevant listening materials.

## 2 Literature review

### 2.1 Listening comprehension challenges in EFL contexts

Listening comprehension has been highlighted as one of the most difficult skills for EFL learners to develop (Richards, 2008). Listening requires real-time processing of rapidly delivered speech with limited opportunities for clarification, unlike reading, where learners can control the pace and revisit text. Compared with writing, reading, and speaking, listening skill involves more interpretation, memorization, comprehension, and evaluation (Al-Khresheh, 2020). Several factors contribute to such listening comprehension difficulties; for instance, linguistic factors, such as unfamiliar vocabulary and complex grammatical structures, can play a major role. Cognitive issues are also fundamental. For example, poor working memory and slower processing speed can influence general comprehension. Listening may also be impaired by emotional aspects such as anxiety and lack of confidence (Hayati, 2009; Vandergrift, 2007).

Other contextual factors further complicate the aforementioned issues in the Saudi context. According to (Mahboob and Elyas 2014), English has historically been taught in Saudi Arabia with a focus on grammar and reading over communicative skills, leaving many learners unequipped for authentic listening tasks. Additionally, the predominant use of Western-centric materials in Saudi EFL classrooms may further complicate the current difficulties by presenting content that lacks cultural relevance to learners' lived experiences, requiring additional cognitive resources to process unfamiliar cultural references while simultaneously managing linguistic demands (Al-Malki, 2018).

### 2.2 Cultural teaching materials in language learning

The theory of schema provides a strong framework for explaining how cultural context affects language comprehension. It suggests that comprehension involves using background knowledge structures (schemata) to help learners process new information (Anderson and Pearson, 1988). Self-determination theory (Ryan and Deci, 2000) adds a motivational dimension to this picture: content that mirrors learners' own experiences and values is better positioned to satisfy the psychological needs for competence and autonomy, thereby encouraging independent and proactive engagement. Combined with schema theory, the two frameworks converge on the prediction that culturally familiar content simultaneously reduces cognitive demands and strengthens the motivational conditions that support agentic participation. When listening materials are based on culturally focused content, it becomes easier for the learner to activate their own schemata. Thus, understanding becomes much easier, and less cognitive load is needed.

Empirical research supports the positive impact of culturally relevant materials on language learning outcomes. Cultural Relevance refers to the degree to which instructional materials and practices reflect learners‘ cultural knowledge, values, and social contexts (Gay, 2018). According to (Sheridan and Condon 2020), EFL learners were more motivated and engaged when reading texts related to their cultures. In a parallel quasi-experimental study, (Diep et al. 2022) found that EFL learners exposed to culture-based instructional materials outperformed control-group peers on reading comprehension, reading motivation, and reading attitude. Similarly, (Kong et al. 2022) found that culturally responsive teaching enhanced teacher-student engagement in rural Chinese EFL settings. In Saudi EFL contexts, materials grounded in learners' local cultural references have been argued to support engagement and identity-related dimensions of learning (Al-Malki, 2018). Literature on culturally responsive teaching has consistently demonstrated positive effects on motivation, engagement, and achievement across various learning settings (Gay, 2018).

Yet other scholars caution against relying solely on culturally familiar resources, arguing that achieving intercultural communicative competence requires exposure to target-language cultures (Sheridan and Condon, 2020). (Ali et al. 2015) state that teaching a language in isolation from its culture makes it harder for students to communicate in any real or meaningful way. They go so far as to say that incorporating culture into English language teaching is not only a nice addition but also an essential part of the learning process. In light of such studies, an optimal approach might involve balancing culturally relevant materials with those from the target culture, providing learners with both the scaffolding of familiar contexts and the enrichment of new cultural perspectives. The present study aligns with this balanced view. Culturally contextualized listening materials are examined not as a replacement for target-culture content but as a complementary scaffold that may support specific dimensions of learner engagement, while exposure to target-culture materials remains essential for the development of intercultural communicative competence.

### 2.3 The concept of multidimensional engagement in language learning

Engagement in language learning has increasingly attracted scholarly attention in educational research. This concept refers to the level of learner engagement in learning processes and in any classroom task (Philp and Duchesne, 2016). It measures the extent to which students demonstrate active involvement while completing assigned activities (Christenson et al., 2012). To develop engaging classes, teaching resources must include clear objectives, measurable outcomes, and be contextualized (Egbert et al., 2021). *Contextualization* here refers to the pedagogical practice of situating learning content within a specific context of interest to the learner in order to support comprehension and engagement (Mazzeo et al., 2003). The engagement of EFL learners in educational activities involves active participation, motivation, commitment, attention, effort, and interest in the learning process (Zare and Derakhshan, 2024).

**Figure 1:**
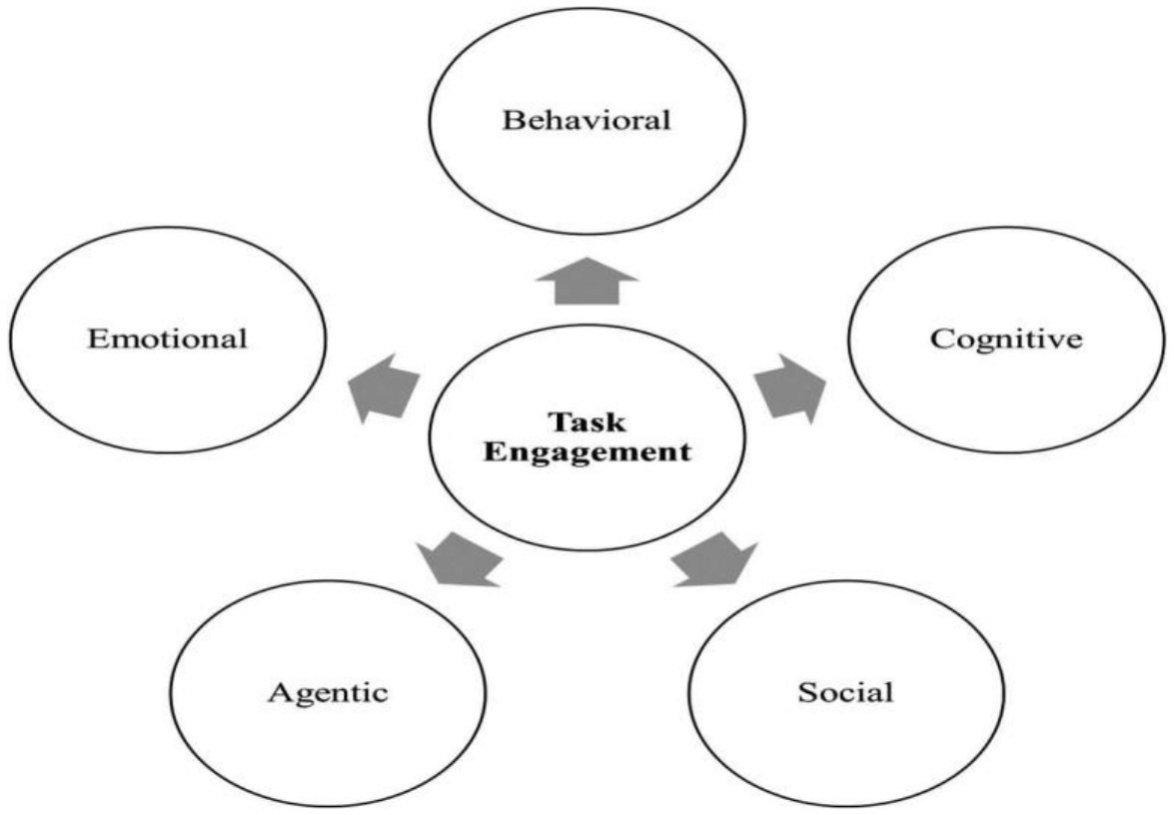
The dimensions of task engagement. Reproduced with permission from (Zare and Derakhshan 2024), License No. 6300111217803. Based on the framework of (Egbert et al. 2021).

Available literature suggests that task engagement takes several forms. (Egbert et al. 2021), as shown in Figure 1, identified five dimensions: behavioral, emotional, cognitive, agentic, and social engagement. First, the behavioral dimension concerns commitment, involvement, initiative, and perseverance in task completion (Skinner et al., 2009). It records observable classroom interaction of learners (Philp and Duchesne, 2016) or energized, directed, and sustained classroom action (Skinner et al., 2009). Some measurable indicators include time spent on the task, verbal input, visual attention patterns, physical behavior, following instructions, and responding to questions (Dörnyei and Kormos, 2000; Oga-Baldwin, 2019; Philp and Duchesne, 2016).

The second component is the emotional engagement, which involves learners' affective participation and psychological connections to learning activities (Sang and Hiver, 2021). This entails positive emotions such as satisfaction, enthusiasm, and curiosity—often referred to as 'task-facilitating' emotions. Besides these constructive emotions, there are other negative emotions—such as tension, apathy, dread, and frustration—classified as 'task-withdrawing' emotions (Reeve, 2012; Shao et al., 2019). Third, the cognitive dimension concerns conscious awareness, focused concentration, intentional reasoning, intellectual work, and metacognition (Dao, 2021; Helme and Clarke, 2001). It is defined as the degree to which learners are attentive, put mental effort into completing tasks, connect existing knowledge, and check their progress in learning (Mitchell and Carbone, 2011).

The fourth component is the agentic dimension, which refers to learners' proactive, intentional, and constructive input in the learning activity (Reeve, 2012). It emphasizes how learners can improve learning and teaching. These include seeking clarification, offering opinions, and expressing preferences regarding materials and teaching methods. These behaviors show how learners function as agents who 'not only react to learning activities, but also proact upon them' (Henry and Thorsen, 2020). Some studies noted that agentic engagement focuses more on classroom-observable behaviors in which learners affectively influence the instruction (Oga-Baldwin, 2019). Lastly, social engagement refers to a learner's willingness to interact and to establish and maintain such interaction (Svalberg, 2009). It has a powerful influence on academic performance and classroom relationships (Moranski and Toth, 2016). This multidimensional framework provides a comprehensive method of analyzing how culturally situated teaching materials affect various aspects of learner engagement beyond standard engagement measures.

### 2.4 Artificial intelligence in learners' engagement

The past few years have seen significant growth in the development of AI technologies for language education. This is due to advances in natural language processing, machine learning, and speech synthesis (Mukhallafi, 2020; Woo and Choi, 2021). The variety of AI uses in language learning includes intelligent tutoring systems, automated writing assessment, adaptive learning environments, conversational practice chatbots, and content generation (Zawacki-Richter et al., 2019). The effects of these AI tools on various aspects of language learning have recently been studied. (Yuan and Liu 2024) found that AI-assisted learning increased EFL learners' engagement, enjoyment, and motivation. On the same note, (Roy and Swargiary 2024) found that AI integration had positive effects on student learning outcomes and attitudes. Yet a systematic review by (Zawacki-Richter et al. 2019) revealed that many AI-related educational studies focus on technology development rather than the potential use of AI in pedagogy.

The recent literature consistently emphasizes AI's ability to promote student engagement. (Huang et al. 2023) were among the first to demonstrate this, showing that learners in an experimental group who received AI-generated personalized video recommendations were more successful and engaged, particularly those with moderate motivation levels. Similar results were reported by (Xu and Li 2024), who found that AI affordances positively predicted classroom engagement among 408 Chinese EFL university students. In another study involving 113 Chinese EFL students, (Wang and Xue 2024) demonstrated that the use of AI tools in EFL classrooms led to higher levels of student engagement. AI has the potential to transform a hybrid education system by supporting learner and teacher autonomy and providing a more interactive learning experience (Almusaed et al., 2023).

LLMs such as ChatGPT, Claude, and Gemini have demonstrated their ability to generate coherent, contextually relevant text across different genres and topics. When used with advanced text-to-speech systems, these technologies can create listening materials that are closer to natural speech in terms of prosody, intonation, and fluency. Implementing AI tools in the classroom can offer students learning experiences that feel more personal, interactive, and responsive. Instead of using the same material the same way, students receive in-the-moment feedback and follow paths that suit their learning styles. AI can also breathe life into traditional course content, turning what used to be boring and one-directional into something students can actively engage with. These kinds of rich, immersive experiences not only hold students' attention but also help learning in a more enjoyable way (Wadhwa et al., 2024). Nevertheless, research examining the pedagogical effectiveness of AI-generated listening materials remains limited, particularly concerning their potential to support culturally responsive teaching.

A more nuanced reading of the literature, however, reveals that the picture is not uniformly favorable. Several recent studies have raised concerns or reported mixed effects. In a meta-analysis of 32 empirical studies, (Deng and Yu 2023) found that although chatbot-based learning yielded medium-to-high gains on overall learning outcomes, it produced negative findings for learning engagement, motivation, and critical thinking. Similarly, (Wu and Yu 2024) meta-analysis of 24 randomized studies cautioned that part of the apparent benefit of AI chatbots may be attributable to novelty effects that diminish over longer interventions. (Crawford et al. 2024), drawing on structural equation modeling with 387 university students, reported a net negative effect on student achievement once social belonging and loneliness were accounted for, suggesting potential displacement effects when AI substitutes for human interaction. (Lin and Chen 2024) further documented that repetitive AI interactions can trigger emotional disengagement and heightened performance anxiety alongside the benefits typically reported. These contrasting findings underscore the need to identify the specific mechanisms through which AI features (such as personalization, real-time responsiveness, or novelty) may shape learner engagement, particularly when those features may operate independently of cultural content.

### 2.5 Research gap and study contribution

The literature review identifies several gaps that this research study aims to address. First, although research has found that culturally focused materials benefit language learning, only a limited number of studies have examined their direct impact on listening comprehension and engagement. Second, AI technologies have potential in language education, though there is scant research on AI-generated culturally contextualized materials and their influence on learners' engagement. Third, the majority of students' engagement research focuses on a single dimension or general measures, without accounting for how the five dimensions of engagement may respond to specific pedagogical interventions. This study investigates how cultural contextualization within AI-generated listening materials affects five dimensions of engagement among Saudi EFL learners, comparing culturally familiar (Saudi) content against target-language (Western) content. The comparison is between cultural contexts, not between AI-generated and human-produced materials. The present study investigates the following research questions:

**RQ1**: To what extent does cultural contextualization in AI-generated listening materials affect Saudi EFL learners' multidimensional engagement (behavioral, emotional, cognitive, agentic, and social)?

**RQ2**: Which dimensions of engagement are most influenced by local culture listening content compared to target-language culture content?

## 3 Method

### 3.1 Participants

The participants involved in this study were all male Saudi medical students in the preparatory program at a Saudi University between 18 to 19 years of age. Most of the credit hours in the preparatory program are allocated to English courses designed to improve students' English proficiency before they progress to the medical track. The participants were native Arabic speakers whose English proficiency was assessed through institutional placement tests at the CEFR B1-B2 level. The placement test used by the institution is the Oxford Placement Test (OPT), which assigns CEFR-aligned proficiency bands. Participants had studied English for an average of 10 years and were enrolled in a required listening and speaking course at the time of data collection.

Although 92 students initiated the study, only participants who completed both listening tasks and both post-assessment surveys (*n* = 78) were included in the final analysis. Completion of both tasks and surveys served as an inclusion criterion. The participants were from three different class groups with assistance from each group's English instructor. The participants were recruited through classroom announcements and voluntary sign-ups. Inclusion criteria include: (a) taking the intermediate English program, (b) being a native Arabic speaker, and (c) placed at CEFR B1–B2. The exclusion criteria excluded students at B2 + levels and those with a bilingual background, defined as regular use of a second language other than Arabic in the home environment, to avoid ceiling effects. Reliability coefficients for the OPT in this specific population were not available. Informed consent was obtained from all participants, who were informed about the purpose of the study, the procedures, and their rights. Anonymity and confidentiality were maintained. It was also made clear to the participants that they could drop out without any penalty.

### 3.2 Instrument

#### 3.2.1 Listening materials

Two listening recordings were developed for this study. Each recording consisted of a 2-min dialogue between two speakers and was created specifically for the research context. Scripts were generated using *ChatGPT 4.0* based on detailed prompts to produce conversational content on similar topics, minimizing content bias. Both contain cultural elements from each country. It shows different cultures that mark the differences of both tasks. The two scripts were reviewed for cultural accuracy, sensitivity, and the avoidance of cultural stereotyping prior to data collection. The full *ChatGPT* prompts and complete dialogue scripts for both Task A and Task B are provided as supplementary materials (Appendix B and C) to support reproducibility and independent evaluation of the cultural content.

The scripts were converted to audio using *Speechify*, a text-to-speech system producing naturalistic-sounding speech approximating conversational register with prosodic parameters (intonation, stress, rhythm) consistent with fluent English delivery. The same text-to-speech engine and comparable voice profiles were used for both Task A and Task B, so that prosodic naturalness and acoustic quality were held constant across conditions. Prosodic consistency in this study served as a methodological control rather than a claim of full communicative authenticity; no formal acoustic or listener-panel validation of the recordings was conducted.

A pilot test was conducted with 10 students drawn from the same preparatory program as the main study participants; pilot participants were matched to the main sample on language background and CEFR proficiency level (B1–B2) and were excluded from the final sample of 78. The pilot procedure was identical to that of the main study: pilot participants listened to both recordings and completed the engagement questionnaire and the 10-item comprehension test for each task. Pilot comprehension accuracy was 86% for Task A and 84% for Task B. Minor adjustments to speech rate and two vocabulary items were made based on pilot feedback before the main study.

Task A (Saudi Culture) was a conversation between two Saudi university students discussing plans to attend a football match, including culturally specific references to Saudi football culture, the Saudi Pro League, the Saudi Ticketing Platform, and local fan traditions. The dialogue incorporated culturally authentic communication patterns and social norms typical of Saudi university students. Task B (Western Culture) was a conversation between two American university students discussing plans to attend an NFL game, including references to American football culture and traditions. The dialogue reflected communication patterns and cultural practices typical of American university students.

The two listening tasks were closely monitored for key features to ensure comparability. Both discussions were rated intermediate (CEFR B1 -B2) when it comes to the range of vocabulary and grammar structure. The speech rate of the two recordings was around 145 words per min. About 2 min were allotted to each recording in which two speakers engaged in an informal dialogue about attending a sports event. Neither recording used idiomatic expressions or vocabulary beyond the intermediate level.

#### 3.2.2 Comprehension questions

To encourage participants to actively engage with the listening content, each listening task was followed by 10 multiple-choice comprehension questions (Appendix D). These questions were used to assess students' literal and inferential knowledge. The 10 items following each listening recording were developed and validated through the following procedure: (a) items were independently reviewed by two EFL instructors with expertise in listening assessment for content validity and CEFR-appropriateness; (b) ambiguous or overlapping items were revised through discussion until consensus was reached and (c) the items were trialed in the pilot test described in Section 3.2.1 (*n* = 10), in which all participants completed the comprehension questions for both recordings; pilot performance was used to identify problematic items and to confirm that the two question sets were of comparable difficulty for the target proficiency level. Formal item-analysis statistics (difficulty and discrimination indices) and inter-rater reliability coefficients for the validation review were not computed. The questions were not used as outcome measures; rather, they ensured that participants were paying attention and comprehending what was being said.

#### 3.2.3 Engagement questionnaire

An adapted version of the Task Engagement Questionnaire by (Zare and Derakhshan 2024) was used to measure the different types of learner engagement. The questionnaire assesses five dimensions of engagement in second-language acquisition and contains 25 questions measured on a five-point Likert scale (1 = Strongly Disagree to 5 = Strongly Agree). Because the original instrument was designed for interactive, classroom-based language tasks, the questionnaire was adapted for the present individual-listening context. Adaptations included changing the response scale from a frequency-based format (1 = Never to 5 = Always) to an agreement-based format (1 = Strongly Disagree to 5 = Strongly Agree) and rewording items to refer specifically to the listening recordings. The behavioral, emotional, and cognitive subscales transferred directly to the listening context with only minor wording changes. The agentic and social subscales, however, retain in their item wording the original instrument's reference to interaction with the teacher and peers (e.g., “During the task, I let my teacher know what I needed and wanted”; “I asked the other students to help me do the tasks”), as shown in Appendix A. Because the present tasks were completed individually, these two subscales should be understood as measuring learners' self-reported orientation toward agentic and social engagement rather than directly observed interactive behavior. This wording limitation, and its consequences for the interpretation of the social engagement results in particular, is addressed in Sections 4.3.5 and 5.1. The full adapted questionnaire is provided in Appendix A. The internal consistency of the adapted 25-item scale in the present study was excellent (Cronbach's α =0.94). Table 1 shows each engagement dimension and a sample item.

**Table 1:** The questionnaire items distribution for each engagement dimension.

Engagement dimension	Sample item
Behavioral (5 items):	“I stayed focused on the listening task and avoided distractions”
Social (5 items):	“I asked the teacher to help me do the listening task.”
Emotional (5 items):	“I felt interested while listening to the task.”
Cognitive (5 items):	“While listening, I tried to connect the ideas in the task with what I already know.”
Agentic (5 items):	“During the task, I asked the teacher questions to help me learn.”

### 3.3 Procedure

The data collection was conducted over 2 weeks during regular class time. The study utilized a within-subjects design, in which all participants completed both the two listening tasks and their post-assessment questionnaires. Participants were drawn from three intact class groups (Group 1, n = 31; Group 2, n = 31; Group 3, n = 30); sampling was non-random and based on classroom-level volunteer enrollment. As described in Section 3.1, 78 of the 92 invited participants completed all study components and were included in the final analysis. Notably, ethical approval was obtained from the institution's Human Subjects Committee prior to data collection.

All participants completed Listening Task A (Saudi culture) in the first week, followed by Listening Task B (Western culture) 1 week later. Immediately after listening to the audio, participants were asked to complete 10 multiple-choice questions to assess their understanding. These questions served to confirm that participants listened carefully to the audio and understood its content. After completing the comprehension questions, participants were presented with the 25-item engagement survey, which aligned with the audio they had just listened to. All sessions were supervised by the researchers to ensure consistency and to answer any inquiries. There was no support offered in terms of content of understanding or responses to surveys to prevent impacting engagement ratings.

## 4 Results

### 4.1 Preliminary analyses

#### 4.1.1 Data screening and descriptive overview

Data analysis was conducted using Jamovi (Version 2.6; The jamovi project, 2024). All 78 participants completed both listening tasks and all questionnaire items, with no missing data. There were no outliers in the descriptive statistics or boxplots; all values fell within 3 SD of the mean. In both tasks, we used Shapiro-Wilk tests to assess the normality of each engagement dimension. All the W values were 0.976 and higher, and all the *p* values were larger than 0.066, which satisfies the assumption of normality in paired-sample *t*-tests (refer to Table 2 to see the detailed values). Because the normality assumption for paired-samples *t*-tests properly concerns the distribution of difference scores rather than each task distribution separately, Shapiro-Wilk tests were also conducted on the difference scores for each engagement dimension. All difference-score distributions were normally distributed (see Table 3). The assumption of normality of difference scores was therefore satisfied for all five paired-samples t*-*tests, supporting the use of parametric analyses throughout.

**Table 2:** Shapiro-Wilk normality test results for all engagement dimensions (per task).

Engagement Dimension	N	Task A (W)	Task B (W)	Interpretation
Behavioral	78	0.976	0.976	Normal distribution (*p* = 0.14)
Emotional	78	0.979	0.979	Normal distribution (*p* = 0.22)
Cognitive	78	0.979	0.979	Normal distribution (*p* = 0.22)
Agentic	78	0.987	0.987	Normal distribution (*p* = 0.60)
Social	78	0.990	0.990	Normal distribution (*p* = 0.81)

**Table 3:** Shapiro-Wilk normality test results for the difference scores (task A – task B).

Engagement dimension (Difference scores)	*W*	*p*
Behavioral (A–B)	0.976	0.140
Emotional (A–B)	0.979	0.219
Cognitive (A–B)	0.979	0.218
Agentic (A–B)	0.987	0.595
Social (A–B)	0.990	0.806

All distributions were normal (all *p* > 0.05).

Cronbach's alpha was used to test the internal consistency of the 25-item engagement questionnaire. The overall scale demonstrated excellent internal consistency (α = 0.94), indicating strong coherence among items measuring the five engagement dimensions. Subscale-level reliability coefficients were also computed and demonstrated acceptable to excellent internal consistency for each dimension: Behavioral (α = 0.82), Emotional (α = 0.92), Cognitive (α = 0.87), Agentic (α = 0.88), and Social (α = 0.89). These coefficients support the use of each subscale as an independent measure in the dimension-level analyses that follow.

#### 4.1.2 Comprehension verification

Comprehension scores were also analyzed to ensure that the participants were engaged with listening content and that potential differences in engagement were not attributable to task comprehension difficulty. Participation in both audio recordings revealed overall adequate comprehension, with a mean accuracy of 82.3% (SD=11.2%) for Task A (Saudi culture) and 80.7% (SD=12.5%) for Task B (Western culture). A Paired-samples *t*-test showed no significant difference in comprehension between the two listening tasks, t (77) = 1.23, *p* = 0.222, indicating equivalent task difficulty and participant engagement with the content.

### 4.2 Descriptive statistics

Table 4 shows descriptive statistics of all five engagement dimensions. The table compares students' responses on the culturally relevant task (Task A: Saudi culture) with those on the target-language culture task (Task B: Western culture).

**Table 4:** Descriptive statistics for engagement dimensions by task.

Engagement dimension	*N*	Task A (Saudi culture)			Task B (Western culture)		
		*M*	*SD*	*Mdn*	*M*	*SD*	*Mdn*
Behavioral	78	3.80	0.90	3.90	3.80	0.90	4.00
Emotional	78	3.34	1.03	3.40	3.05	1.07	3.00
Cognitive	78	3.25	1.06	3.20	3.18	1.04	3.20
Agentic	78	2.96	0.93	3.00	2.54	1.07	2.50
Social	78	2.47	1.03	2.40	2.34	1.15	2.20

All measures were rated on a 5-point likert scale (1 = strongly disagree; 5 = strongly agree). M, mean; SD, standard deviation; Mdn, median.

The descriptive statistics indicate meaningful variations across engagement dimensions. The mean behavioral engagement score was the same across both tasks (M=3.80). The greatest differences were found in emotional and agentic involvement with the culturally relevant content, with higher scores. Agentic engagement showed the largest mean difference (0.42 points on the 5-point scale), suggesting that this dimension was more responsive to cultural contextualization than the others.

### 4.3 Primary analyses: paired-samples *t-*tests

To test the hypothesis that statistically significant differences were observed between the engagement dimensions in two listening exercises, paired-samples *t*-tests were performed for each listening exercise. This within-subject design provides greater statistical power by controlling for individual differences in baseline engagement, English proficiency, and listening ability. Cohen's d effect-size benchmarks were applied per (Cohen 1988): small (0.20), medium (0.50), and large (0.80). Table 5 presents the complete results of the paired-samples *t*-tests comparing all five engagement dimensions across the two tasks. Because five engagement dimensions were tested simultaneously, a Bonferroni correction for multiple comparisons yields an adjusted alpha of.05 / 5 = 0.010. The agentic engagement finding (*p* = 0.008) remains significant under this corrected threshold, though the margin is narrow and the result should be interpreted with appropriate caution. The subsections below elaborate more on each dimension.

**Table 5:** Paired-samples t-tests comparing engagement across tasks.

Engagement Dimension	*t*	*df*	*p*	Cohen's *d*	95% CI	**Mean Diff**.
Behavioral	0.04	77	0.971	0.004	[−0.28, 0.29]	0.005
Emotional	1.86	77	0.066	0.211	[−0.02, 0.60]	0.290
Cognitive	0.42	77	0.674	0.048	[−0.27, 0.41]	0.072
Agentic	2.70	77	0.008^**^	0.306	[0.11, 0.72]	0.415
Social	0.83	77	0.408	0.094	[−0.18, 0.44]	0.131

^**^p < 0.01 (statistically significant at the 0.01 level).

#### 4.3.1 Behavioral engagement

There was no statistically significant difference in behavioral engagement between Task A (*M* = 3.80, *SD* = 0.90) and Task B (*M* = 3.80, *SD* = 0.90), t (77) = 0.04, *p* =0.971, Cohen's *d* = 0.004, 95% CI [−0.28, 0.29]. The effect size was non-significant, indicating that there was practically no difference in the level of self-reported behavioral involvement between participants using culturally relevant and target-language materials.

#### 4.3.2 Emotional engagement

The trend of emotional engagement expressed a greater difference between culturally relevant content (Task A: *M* = 3.34, SD = 1.03) and the Western content (Task B: *M* = 3.05, *SD* = 1.07), but that difference was not statistically significant, t (77) = 1.86, *p* =0.066, Cohen's d = 0.21, 95% CI [−0.02, 0.60]. The effect size was small (d = 0.21).

#### 4.3.3 Cognitive engagement

The difference between cognitive engagement in Task A (M = 3.25, SD = 1.06) and Task B (M = 3.18, SD = 1.04) was not statistically significant, t (77) = 0.42, *p* = 0.674, Cohen's d = 0.048, 95% CI [-0.27, 0.41]. Self-reported cognitive engagement was therefore similar across the two tasks. This pattern is consistent with the possibility that cognitive engagement was associated more with the linguistic and cognitive demands of the tasks, which were comparable, than with cultural familiarity, although the present design does not allow this to be tested directly.

#### 4.3.4 Agentic engagement

A statistically significant difference was observed in agentic engagement between Task A (M = 2.96, SD = 0.93) and Task B (M = 2.54, SD = 1.07), t (77) = 2.70, *p* = 0.008, Cohen's d = 0.31, 95% CI [0.11, 0.72]. The 0.42-point mean difference on the 5-point scale represents a small but statistically significant advantage in agentic engagement for the culturally relevant materials. Descriptively, participants reported higher levels of autonomy, ownership, and proactive involvement on the culturally relevant task than on the target-culture task. These self-reported differences are consistent with greater perceived empowerment, motivation, and control over learning in the culturally relevant condition; given the design, they are interpreted as associations rather than as causal effects of cultural content.

#### 4.3.5 Social engagement

No statistically significant difference was detected in social engagement between Task A (*M* = 2.47, *SD* = 1.03) and Task B (*M* = 2.34, *SD* = 1.15), t (77) = 0.83, *p* = 0.408, Cohen's d = 0.094, 95% CI [−0.18, 0.44]. As noted in Section 3.2.3, the social engagement items retain the original instrument's wording referring to interaction with the teacher and peers (e.g., asking the teacher or other students for help), even though the listening tasks in this study were completed individually. This creates an important interpretive caveat: because the task format offered little or no opportunity for the peer-and-teacher interaction these items describe, low social engagement scores are an expected by-product of the task design rather than evidence that culturally relevant content failed to support social engagement. The relatively low scores in both conditions (M < 2.50) are therefore most plausibly attributable to the individual, non-interactive nature of the task itself. For this reason, the social engagement results in this study should be interpreted with particular caution, and the social dimension may be better assessed in future work using collaborative or discussion-based listening formats, or using items reworded to suit individual tasks. Other contributing factors may include the brevity of the dialogues (2 min each), the absence of visual cues, and the fact that the speakers were unfamiliar AI-generated voices.

### 4.4 Summary of key findings

The response to Research Question 1: What is the impact of cultural contextualization on multidimensional engagement? revealed a subtle, dimension-specific effect. Behavioral, emotional, cognitive, and social engagement did not differ significantly between culturally relevant and target-language materials. The only dimension showing a statistically significant difference was agentic engagement, where culturally relevant materials were associated with a small increase (*d* = 0.31). This single, small effect does not represent a broad enhancement of multidimensional engagement. In response to Research Question 2: Which dimensions are most influenced by cultural relevance? The highest and only statistically significant effect was agentic engagement in response to cultural relevance. This suggests that cultural relevance may support, though not consistently, the dimensions of engagement most closely tied to learner autonomy and agency. However, because both tasks were AI-generated, the present design isolates the effect of cultural content within an AI-generated medium; it cannot, however, separate the contribution of cultural content from that of the AI-generated nature of the materials. Any engagement difference observed between the two tasks is therefore interpreted with this constraint in mind.

## 5 Discussion

The main finding showed a significant but small increase in agentic engagement when learners are exposed to culturally relevant materials (*p* = 0.008, *d* = 0.31). This result should be treated as preliminary because of the order effect and multiple-comparisons issues discussed in Section 4. Still, it suggests that AI-generated materials connected to learners' cultural context may help students feel more involved and responsible for their learning. Since agentic engagement is linked to learners' initiative, autonomy, and active contribution, culturally relevant materials may support this type of engagement by making learning feel more personally meaningful.

In terms of self-determination theory, cultural relevance may help support the psychological need for autonomy (Ryan and Deci, 2000). Learning activities may become more self-directed when learning resources reflect learners' experiences and values, though this interpretation is inferred from the engagement ratings rather than directly tested in the present study. Pedagogically, agentic engagement is widely regarded as valuable because autonomous learners tend to persist through challenges and develop intrinsic motivation (Benson, 2011).

The absence of significant differences in the other four engagement dimensions may be explained in several ways. First, both listening tasks appeared to be similarly accessible to students, as comprehension scores did not differ significantly between the two conditions. This suggests that the design and level of the materials may have allowed students to understand both tasks, even when the cultural context was less familiar. Although schema theory suggests that familiar content can support comprehension, the present findings indicate that cultural familiarity did not lead to a significant comprehension advantage in this study. Second, emotional engagement showed a near-significant pattern (*p* = 0.066), with a small effect size (d = 0.21). Third, social engagement may have been limited by the individual nature of the listening task and the lack of peer interaction. Because both conditions produced low social engagement scores (M < 2.50), the task format may have restricted opportunities for cultural relevance to influence this dimension.

Enhanced agentic engagement, as found, is consistent with prior studies on culturally focused teaching. (Kong et al. 2022) found that culturally focused teaching in a rural Chinese EFL setting increased teacher and student engagement; the current research extends this to student engagement specifically in a Saudi context and identifies agentic engagement as the key dimension affected. Similarly, (Hua and Thao 2024) associated cultural awareness with learner autonomy among Vietnamese EFL learners, which supports the present study's finding of growing agentic engagement.

The non-significant trend in emotional engagement (*p* = 0.066, *d* = 0.21) is partly consistent with studies that find culturally familiar content to be a stronger trigger of affective responses (Sheridan and Condon, 2020). However, this difference did not reach statistical significance and the small effect size (*d* = 0.21) should be interpreted as a weak, uncertain relationship that requires replication before any substantive conclusion is drawn.

The application of AI-generated materials in this paper is added to a list of studies that revolve around AI applications in language education (Mukhallafi, 2020; Woo and Choi, 2021; Yuan and Liu, 2024). Although previous research has focused on the effects of AI on overall engagement and motivation, the current study provides more detailed data, revealing that AI-generated materials can be culturally relevant and directed toward specific areas of learner engagement.

### 5.1 Limitations

Several caveats apply to the interpretation of these findings. Regarding task order, all participants completed the Saudi-culture task in the first week and the Western-culture task in the second week. Separating the tasks by 1 week was intended to reduce carryover effects such as fatigue and immediate contrast. However, because the order was not counterbalanced across participants, sequence-related influences, including greater familiarity with the procedure by the second task, cannot be fully ruled out as partial contributors to the observed agentic engagement difference. A second concern is that the participant sample is highly restricted: all male Saudi medical preparatory students at a single institution, in the 18–19 age range, all native speakers of Arabic. This sharply limits external validity and generalizability to female learners, other age groups, other proficiency levels, other disciplinary contexts, mixed-gender classrooms, and other EFL settings. Statements throughout the manuscript should be read with this restriction in mind.

A third concern, closely related to the fixed task order, is the possibility of bias from topic interest or prior familiarity. The two tasks differed not only in cultural framing (Saudi vs. Western) but also in specific topical content (e.g., Saudi football vs. the NFL and Western media references). The study did not measure participants' pre-existing interest in, or prior exposure to, either topic. It is therefore possible that differences in engagement reflect differential interest in the subject matter or greater everyday familiarity with local sporting and media content, rather than cultural contextualization as such. Because globalized media mean that Saudi students may also have considerable exposure to Western sports and entertainment, the assumption that the Western task was genuinely “less familiar” was not directly verified. Future studies should measure topic interest and prior cultural exposure directly, so that these factors can be modeled or controlled rather than left confounded with cultural relevance. A fourth concern relates to the measurement of comprehension. The comprehension questions were reviewed by two instructors and trialed in the pilot, but formal item-analysis statistics (difficulty and discrimination indices) and inter-rater reliability for the review were not computed; the comprehension data should therefore be read only as a coarse check that participants attended to the audio, not as a validated measure of listening proficiency.

Behavioral observation, learning-platform analytics, or other objective indicators were not used to corroborate the questionnaire data. Combining self-report with one of these external sources is therefore a worthwhile direction for replication studies. Finally, an a priori power calculation was not conducted; with n = 78, the present study had limited sensitivity to detect small between-condition differences, which should be borne in mind when interpreting the non-significant results.

## 6 Conclusion

This study offers preliminary, exploratory evidence that AI-generated, culturally contextualized listening materials are associated with higher agentic engagement among Saudi EFL students. **The effect was small and limited to this single dimension; no significant differences emerged for behavioral, emotional, cognitive, or social engagement**. In the present design, AI functioned as a production technology operating under researcher-directed prompting rather than as an independent cultural agent; the cultural design of the materials was driven by human pedagogical intent and curation. The within-subject design found no significant differences in behavioral, social, emotional or cognitive engagement between culturally relevant (Saudi) and target-culture (Western) tasks, likely because both tasks were well matched. However, students exposed to culturally familiar content reported **a small but statistically significant increase in agentic engagement (*d*** **=** **0.31)**, consistent with the idea that learners may be more proactive and participatory as agentic engagement involves the constructive contributions learners make to the flow of instruction.

These findings have implications for pedagogy and educational technology. Instructors can use AI to efficiently produce culturally relevant listening materials that may support learner autonomy and participation. Specifically, the present findings support a narrow practical claim: AI can be a practical tool for generating culturally contextualized listening content, and such content may be associated with modest increases in self-reported agentic engagement. Developers of language learning platforms should therefore include features that allow teachers to customize content to students' cultural backgrounds, ensuring materials resonate with learners and support their agency. Moreover, the positive role of culturally responsive teaching in improving engagement and learning has been documented in prior work.

The study also raises questions for future inquiry. Our participants were intermediate-level medical students at a single Saudi university; future research should include learners from varied proficiency levels, ages, and cultural backgrounds. We also compared only Saudi and Western cultural content; exploring other cultural comparisons would enrich our understanding of contextual effects. Combining self-report measures of engagement with performance or retention data could reveal whether the observed association between culturally contextualized content and self-reported agentic engagement translates into measurable learning gains. Comparative studies of different AI tools (e.g., ChatGPT, Gemini and other large language models) and the role of prior experience with AI would help identify best practices for generating engaging materials. Mixed-methods designs incorporating brief post-task qualitative reflections, stimulated-recall interviews, or focus-group data would also help clarify why agentic engagement responded to cultural contextualization while other dimensions did not.

Two further considerations regarding AI's role in this study warrant explicit discussion. First, since both tasks were produced using the same AI tools and voice settings, the study cannot determine whether the engagement patterns observed reflect the cultural content of the materials, the AI-generated nature of the audio, or both. A 2 × 2 design crossing materials origin (AI-generated vs. human-produced) with cultural relevance (local vs. target-culture) is recommended for future research to isolate these effects. Second, the present results should not be interpreted as evidence that AI automatically produces culturally resonant materials. The cultural authenticity of the scripts depended entirely on the design of the prompts and on post-generation review by researchers familiar with the target cultural contexts, not on the AI system itself. AI-generation should therefore be treated as a starting point requiring structured cultural review, not as a self-sufficient pipeline for culturally responsive materials.

Aside from the design considerations specific to the present study, AI-generated learning content carries practical concerns that any classroom adoption needs to plan for. Outputs can occasionally include factual inaccuracies, particularly when describing local cultural details, so teachers should screen any AI-produced material before use with students. AI systems can also flatten cultural variation into oversimplified or stereotypical representations, treating “Saudi” or “Western” culture as if it were a single uniform category. Therefore, scripts should be reviewed by someone familiar with the target community. The quality of what AI produces is also strongly tied to how the prompt is written, meaning that designing effective prompts is itself a learned skill rather than a guaranteed outcome of using the technology. Finally, intellectual-property questions, data-privacy considerations, and reliance on commercial platforms remain open practical issues that schools and universities need to weigh when integrating such tools.

## Data Availability

The raw data supporting the conclusions of this article will be made available by the authors, without undue reservation.
