# Supplementary material for: Can AI-generated culturally contextualized listening materials enhance agentic engagement? An exploratory study with Saudi EFL students
Source: Front Psychol. 2026 Jul 9;17:1861868. doi: 10.3389/fpsyg.2026.1861868 (PMC13391903; doi:10.3389/fpsyg.2026.1861868)
Supplement: Supplementary file 1 [file Supplementary_file_1.docx]

### Appendix A: Engagement Questionnaire Items

**Instructions**: Please rate your agreement with each statement about the listening activity you just completed.

**Scale**: 1 = Strongly Disagree, 2 = Disagree, 3 = Neutral, 4 = Agree, 5 = Strongly Agree

**Behavioral Engagement**

1. I tried to do more than what was necessary to do the listening task well

2. I stayed focused on the listening task and avoided distractions

3. I spent as much time as necessary to complete the listening task

4. I worked as hard as I could to complete the listening task

5. I tried to actively engage myself in the listening task

**Emotional Engagement**

6. Listening to the audio was fun

7. I felt interested while listening to the task

8. Listening to the audio increased my curiosity

9. I felt enjoyable when listening to the audio

10. I felt enthusiastic when doing the task

**Cognitive Engagement**

11. While listening, I tried to explain the key concepts in my own words

12. While listening, I was summarizing the information in my own words

13. While listening, I tried to connect the ideas in the task with what I already know

14. While listening, I tried to generate examples to help me understand them better

15. While listening, I repeated the contents and asked myself questions about them

**Agentic Engagement**

16. During the task, I let my teacher know what I needed and wanted

17. During the task, I let my teacher know what I was interested in

18. During the task, I expressed my preferences and opinions

19. During the task, I asked the teacher questions to help me learn

20. During the task, when I needed something, I asked the teacher for it

**Social Engagement**

21. I asked the teacher to help me do the listening task

22. I asked the other students to help me do the tasks

23. It was important for me to communicate with the teacher while doing the tasks

24. It was important for me to communicate with the other students while doing the tasks

25. To make sure I did the exercises correctly, I asked the teacher to give feedback

### Appendix B: Listening Passage Transcripts

#### **Task A (culturally relevant): Saudi Football Derby (Al-Hilal vs. Al-Nassr)**

**Khalid:**

“Hey, Abdullah! Did you hear about the big Al-Hilal and Al-Nassr derby this weekend? It’s going to be at Kingdom Arena Stadium in Riyadh. I can’t wait to see Cristiano Ronaldo and Aleksandar Mitrović go head-to-head. It’s going to be intense!”

**Abdullah:**

“Absolutely, Khalid! I’ve been counting down the days. Al-Nassr has been on fire this season, and with Ronaldo leading the attack, I think we have a real shot at winning. But I know Al-Hilal won’t make it easy for us. What do you think?”

**Khalid:**

“Well, Al-Hilal has a solid defense, and Mitrović has been scoring consistently. I think it’s going to be a close match. But honestly, I’m just excited to be there and experience the atmosphere. The energy at Kingdom Arena is always electric, especially for these derbies.”

**Abdullah:**

“Same here! Have you bought your ticket yet? I checked the Saudi Ticketing Platform yesterday, and they still have some good seats available. The regular tickets are 100 riyals, premium seats are 250, and VIP is 500. I’m thinking of going for the premium seats. What about you?”

**Khalid:**

“I was thinking the same thing! Premium seats give you a great view without breaking the bank. Let’s book them together. Do you want to meet up before the match, maybe grab something to eat near the stadium?”

**Abdullah:**

“That sounds like a plan! The match kicks off at 8 PM, so let’s aim to get there by 6 PM. That way, we can soak in the pre-game atmosphere. The area around the stadium will be packed with fans wearing blue and yellow. It’s going to be amazing!”

**Khalid:**

“Perfect! I’ll book the tickets tonight on the Saudi Pro League website. And after the match, we can grab some food near the stadium and talk about the game. I’m sure it’s going to be unforgettable.”

**Abdullah:**

“Definitely! I’ll bring my Al-Hilal scarf, and you better bring your Al-Nassr jersey. Let’s make this a day to remember. See you on Saturday, Khalid!”

**Khalid:**

“See you then, Abdullah! May the best team win!”

#### **Task B (culturally irrelevant): American Football Game (Dallas Cowboys vs. Chicago Bears)**

**Mike:**

“Hey, Jake! Are you ready for the Cowboys versus Bears game this Sunday? It’s going to be huge! Dak Prescott has been playing like a beast this season, and I think Dallas is going to dominate. What do you think?”

**Jake:**

“Well, Mike, I hate to break it to you, but the Bears are looking strong too. Justin Fields has been improving every game, and our defense is solid. I think it’s going to be a close one, but Chicago is going to pull through. You Cowboys fans always get too confident!”

**Mike:**

“Ha! We’ll see about that. Have you grabbed your ticket yet? I checked online, and there are still some decent seats available. The nosebleeds are around $50, but if you want a better view, the mid-level seats are about $120, and the club seats are $250. I’m thinking of going for the mid-level. What about you?”

**Jake:**

“Same here. The mid-level seats are a good deal, and you get a solid view of the field. Let’s book them together. I’ll meet you at the stadium. What time should we get there?”

**Mike:**

“The game starts at 3 PM, so let’s aim to get there by 1 PM. That way, we can enjoy the pre-game vibe. Fans start tailgating in the parking lot by noon, so we can grab some food, check out the fan zone, and maybe even take a few photos with other Cowboys fans.”

**Jake:**

“Sounds like a plan. I’ll meet you by the main entrance around one o’clock, then. I’m bringing my face paint and Bears flag—got to represent Chicago loud and clear!”

**Mike:**

“Ha! I’ll be ready to counter with my Cowboys hat and jersey. Just so you know, Dallas has some of the best food vendors in the NFL. They’ve got barbecue, tacos, even some Texas-style brisket nachos that you’ve got to try. But don’t be surprised if the Cowboys’ cheers are drowning out your Bears chants.”

**Jake:**

“Challenge accepted! And after the game, we should hit up that sports bar nearby to watch the post-game highlights and keep the fun going. I’ll need something to celebrate our win—or to cheer me up if we’re unlucky.”

**Mike:**

“Deal! Let’s make this official, then. I’ll book the tickets, and you can pay me back. I can’t wait to see Dak take down your Bears defense. This game is going to be epic!”

**Jake:**

“You’re on! And when the Bears win, I’ll make sure you know that Chicago pride is unbeatable. Alright, let’s do this! Book those tickets, and I’ll see you at one.”

### Appendix C: AI Generation Prompt

The following prompt was used with ChatGPT-4 to generate both listening passages:

*"****Create two 2-minute listening dialogues for B1-B2 EFL learners, focusing on passionate conversations between fans of rival sports teams. The dialogues should be lively, casual, and culturally relevant, including references to well-known players and terms appropriate to each sport.***

*1.* ***Saudi Derby****: Two football (soccer) fans—one supporting Al-Hilal and the other supporting Al-Nassr—discuss their teams’ chances in the upcoming match. They mention players like Cristiano Ronaldo (Al-Nassr) and Aleksandar Mitrović (Al-Hilal). They talk about plans to buy tickets, meet up, and watch the game together, with some playful rivalry.*

*2.* ***American Derby****: Two NFL fans—one supporting the Dallas Cowboys and the other supporting the Chicago Bears—talk about their teams’ big game. They mention players like Dak Prescott (Cowboys) and Justin Fields (Bears). The conversation includes plans for tailgating, buying tickets, and meeting up before the game, with friendly competition."*

### Appendix D: Sample Comprehension Questions

**Task A (Saudi Football Derby) - Sample Questions:**

1. Why does Abdullah think Al-Nassr will win the match?

a) Because Al-Hilal’s team is weak.

b) Because Cristiano Ronaldo is playing for Al-Nassr.

c) Because Al-Nassr has a good defense.

d) Because Al-Hilal is not prepared.

2. Where can Khalid and Abdullah buy tickets for the match?

a) At the stadium.

b) From the Saudi Ticketing Platform or the Saudi Pro League website.

c) At a local store.

d) They don’t need tickets; the game is free.

3. What are the prices of the tickets for the match?

a) Regular tickets are 100 riyals, premium tickets are 450 riyals, and VIP tickets are 500 riyals.

b) Regular tickets are 100 riyals, premium tickets are 250 riyals, and VIP tickets are 600 riyals.

c) Regular tickets are 100 riyals, premium tickets are 300 riyals, and VIP tickets are 400 riyals.

d) Regular tickets are 100 riyals, premium tickets are 250 riyals, and VIP tickets are 500 riyals.

4. What kind of tickets do Khalid and Abdullah decide to buy?

a) VIP tickets.

b) Premium tickets.

c) Regular tickets.

d) They decide not to buy any tickets.

5. Where is the match taking place?

a) At the King Saud University Stadium.

b) At the Saudi National Stadium.

c) At Kingdom Arena Stadium in Riyadh.

d) At the Al-Nassr Club Stadium.

**Task B (American Football Game) - Sample Questions:**

1. Who does Mike think will win the game?

a) The Chicago Bears

b) The Dallas Cowboys

c) Neither team

d) He doesn’t say

2. What is the price range for mid-level seats?

a) Around $50

b) Around $120

c) Around $250

d) Around $300

3. What time do Mike and Jake plan to arrive at the stadium?

a) 12 PM

b) 1 PM

c) 2 PM

d) 3 PM

4. What American football tradition is mentioned in the conversation?

a) Halftime shows

b) Tailgating

c) Victory parades

d) Pre-game concerts

5. What does Mike say about Dallas stadium food?

a) It’s expensive

b) It’s the best in the NFL

c) It’s limited

d) It’s only barbecue
